# Natural variation in *Drosophila* shows weak pleiotropic effects

**DOI:** 10.1186/s13059-022-02680-4

**Published:** 2022-05-16

**Authors:** Eirini Christodoulaki, Viola Nolte, Wei-Yun Lai, Christian Schlötterer

**Affiliations:** 1 Institut für Populationsgenetik, Vetmeduni Vienna, 1210 Vienna, Austria; 2 Vienna Graduate School of Population Genetics, Vienna, Austria

**Keywords:** *Drosophila*, Experimental evolution, Pool-Seq, Polygenic adaptation, Trait optimum, Pleiotropy, Dominance

## Abstract

**Background:**

Pleiotropy describes the phenomenon in which a gene affects multiple phenotypes. The extent of pleiotropy is still disputed, mainly because of issues of inadequate power of analyses. A further challenge is that empirical tests of pleiotropy are restricted to a small subset of all possible phenotypes. To overcome these limitations, we propose a new measurement of pleiotropy that integrates across many phenotypes and multiple generations to improve power.

**Results:**

We infer pleiotropy from the fitness cost imposed by frequency changes of pleiotropic loci. Mixing *Drosophila simulans* populations, which adapted independently to the same new environment using different sets of genes, we show that the adaptive frequency changes have been accompanied by measurable fitness costs.

**Conclusions:**

Unlike previous studies characterizing the molecular basis of pleiotropy, we show that many loci, each of weak effect, contribute to genome-wide pleiotropy. We propose that the costs of pleiotropy are reduced by the modular architecture of gene expression, which facilitates adaptive gene expression changes with low impact on other functions.

**Supplementary Information:**

The online version contains supplementary material available at 10.1186/s13059-022-02680-4.

## Background

Pleiotropy describes the phenomenon that a single gene affects multiple phenotypes. Because of its importance to understand genetic disorders, evolutionary responses and targeted genetic manipulations, pleiotropy has been the focus of numerous theoretical and empirical studies. Remarkably, two different views on the ubiquity of pleiotropy have emerged. The concept of universal pleiotropy is deeply rooted in quantitative genetics and reasons that pleiotropy must be wide-spread as the number of traits is too large to have a private set of contributing loci [1, 2]. This simple and intuitive consideration is well-supported by experiments documenting the occurrence of substantial pleiotropy [3, 4]. This view was challenged by a series of empirical studies showing an L-shaped distribution of pleiotropy with most genes affecting very few traits and only a small number of genes being truly pleiotropic [5–7]. Despite highly consistent results with different experimental approaches, QTL mapping and gene knockouts, these results remain controversial [8].

The degree of pleiotropy has important consequences for polygenic adaptation. Strong pleiotropic effects for many genes imply that allele frequency changes in response to selection are either not possible or come with considerable cost [9]. Low pleiotropy, on the other hand, facilitates adaptation. Despite this central role of pleiotropy, very little is known about the costs arising from the pleiotropic effects of selected alleles during adaptation processes.

Here, we propose a new approach to quantify pleiotropy, which specifically accounts for two limitations of previous empirical studies, the restriction of analyzing a limited number of traits and the detection limits based on single generation phenotyping. Rather than focusing on the phenotypic impact of a molecular variant on a specific set of traits, we quantify pleiotropy by the fitness cost imposed by changing the frequency of alleles at pleiotropic loci.

We take advantage of experimental evolution to change allele frequencies of multiple loci in response to a new environment. A recent study combining experimental evolution with whole genome re-sequencing (evolve-and-resequence, E&R [10]) identified 99 selected haplotype blocks in 10 replicate *Drosophila simulans* populations exposed to the same hot temperature regime [11]. Unlike the phenotypic response, which converged to a new optimum in all 10 replicates, the underlying genetic changes were quite distinct - different subsets of the 99 selected haplotypes responded in the 10 replicates.

The frequency change of the 99 selected haplotypes reflects the combined effect of fitness advantages from shifting the selected trait closer to its new optimum and cost of pleiotropy arising from changing other phenotypes towards a non-favored direction. Since replicate populations used different sets of loci to approach the new trait optimum imposed by the temperature regime in the laboratory, different pleiotropic costs must have occurred in each replicate. To detect and quantify the pleiotropic costs, we performed controlled admixture experiments between evolved replicate populations. In a mixture of the two populations recessive pleiotropic effects are (partially) masked in heterozygous individuals. If the mixture is imbalanced and pleiotropic effects are not fully dominant, adaptive alleles from the two populations experience different pleiotropic costs due to their frequency in the mixed population (Table S1, S2). It is expected that beneficial alleles from the immigrant population experience a (temporary) fitness advantage after the mixture relative to the alleles from the other population. After mixture, this results in stronger allele frequency increase of alleles beneficial in the immigrant population than those from the recipient population. This expectation is confirmed by computer simulations (Figure S1).

**Fig. 1:**
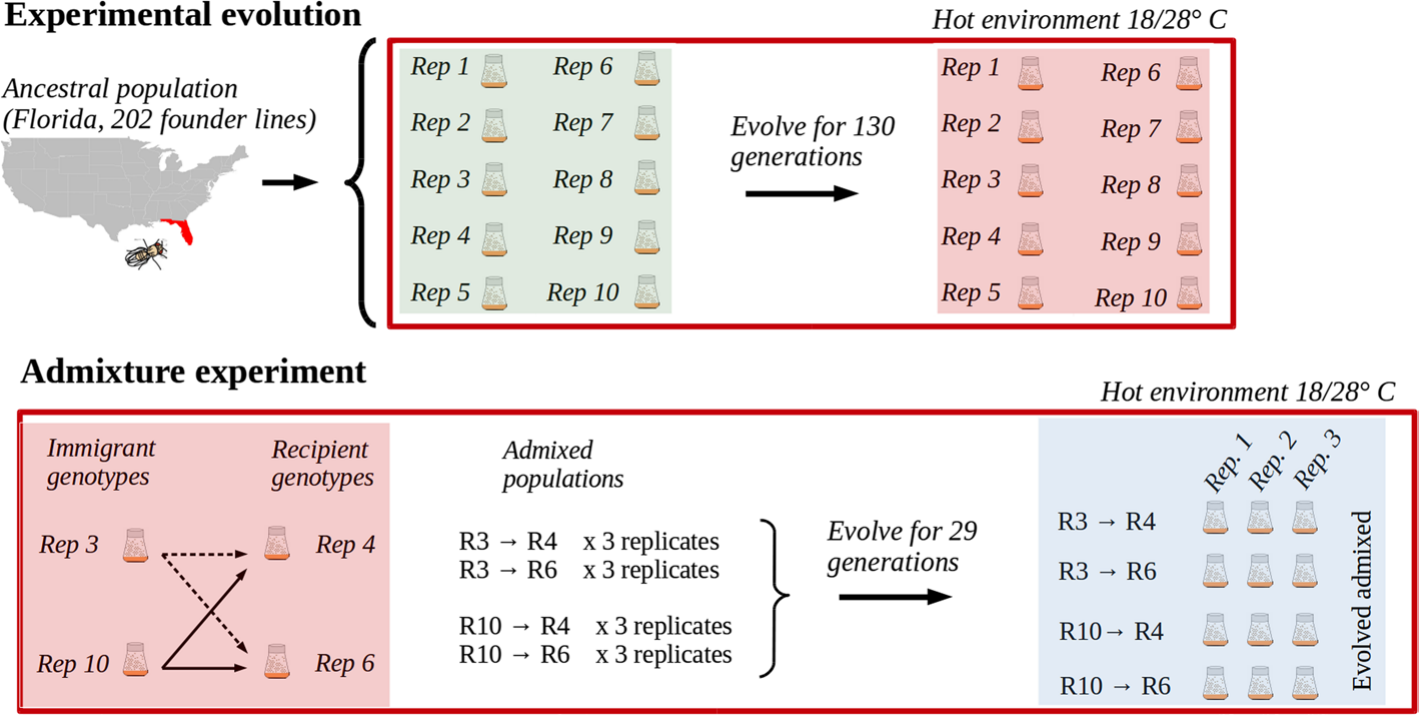
Experimental design: 10 replicate populations were founded by 202 isofemale lines from a natural *Drosophila simulans* population collected in Florida and maintained at a constant population size (1250 flies) with non-overlapping generations in a hot environment (red) fluctuating between 18 and 28 °C on a 12/12 h cycle. At generation 110 four replicates of the evolved populations were used to generate imbalanced pairwise mixed populations (3 replicates for each combination) and maintained in the same hot environment (18°/28°C) for 29 generations (blue). Pool-Seq of the admixed populations at generation F1, F20 and F29 and the parental populations (at generation F110, F120, and F130) was used to estimate genome-wide allele frequencies

To test the impact of pleiotropy, we performed controlled and replicated admixture experiments between replicates with different amount of differentiation and assess their selection response after 30 generations (Fig. 1). Our results confirm the presence of pleiotropic load, but also demonstrate that the effects are rather weak relative to the fitness gain experienced during the adaptation to the novel temperature environment.

## Results

**Fig. 2:**
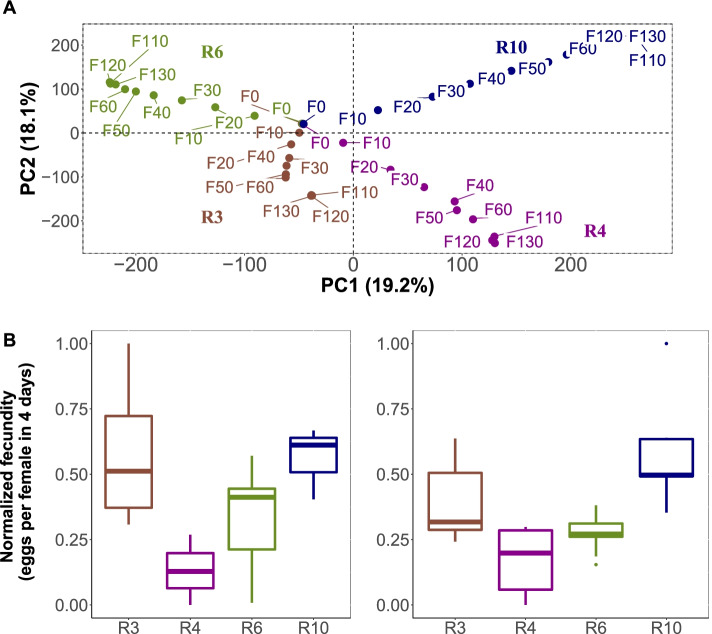
Genomic and phenotypic evolution of the four founder replicates suggest that the evolved populations have approached the trait optimum of the new, hot environment. **A** The Principal Component Analysis of all SNPs shows that the divergence of the 4 replicates slows down at later generations. The absence of pronounced allele frequency changes from generation F110-130 suggests that all populations have approached trait optimum and selection does not cause further allele frequency changes. **B** The relative fecundity of the four replicates remains constant at later time points: F103 on the left F138 on the right panel. Because fecundity increased in all replicates [11], this suggests that the replicates have reached trait optimum, but the fitness component fecundity differs slightly between them. Because different assaying protocols were used for the two time points (M&M) we rescaled the fecundity measurements using the min-max normalization such that 1 is the highest number of eggs observed and 0 is the lowest number of eggs

We used four replicate *D. simulans* populations, which had been exposed for more than 100 generations to a new, hot laboratory environment. A principal component analysis (PCA) illustrates that four replicate populations diverged across time during the experiment. The allele frequency changes were most pronounced at the beginning of the experiment (i.e. after generation 0) and continuously decreased such that generations 110-130 were almost indistinguishable (Fig 2A). The phenotypic response was also very strong, but unlike the genomic response, highly convergent [11]. Only minor differences could be detected in fecundity between the evolved replicates which persisted even after additional 35 generations (Fig 2B). Similar to the limited allele frequency changes between generation 110 and 130, the maintenance of the relative fecundity of the replicates across 35 additional generations is consistent with all populations having approached the same trait optimum, but the measured fitness component differing slightly between them because they used different loci to approach trait optimum.

The proposed test for pleiotropy rests on the idea that different replicates used alternative sets of genes to adapt to the same trait optimum. When the beneficial alleles have pleiotropic side effects, replicate populations will experience pleiotropic costs from different loci. We reasoned that populations which use a similar set of loci to adapt, also share more pleiotropic effects than populations relying on many different loci. We selected therefore two pairs of populations, one highly differentiated and the other one more similar to each other (Table 1). The proposed test of pleiotropy makes two predictions under the assumption that the pleiotropic effects are not fully dominant: 1) low frequency immigrant genotypes will experience a fitness advantage because their pleiotropic effects are masked in heterozygous individuals. For alleles at a higher frequency this is not the case. 2) the advantage will be more pronounced for highly diverged population pairs than for less diverged ones.

**Table 1:**
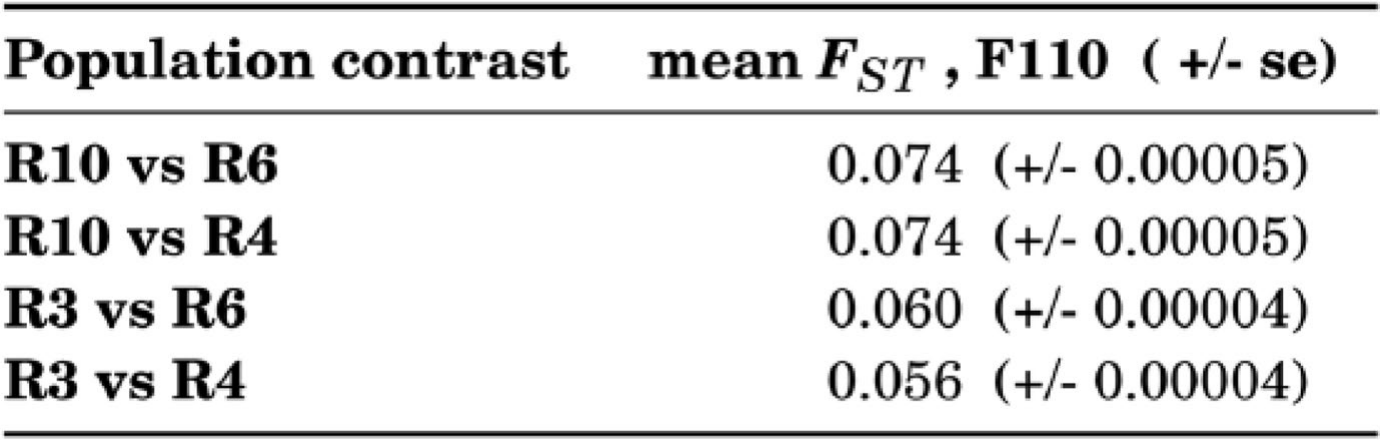
Mean *F*_*ST*_ between the parental populations at the time of admixture (generation 110)

**Fig. 3:**
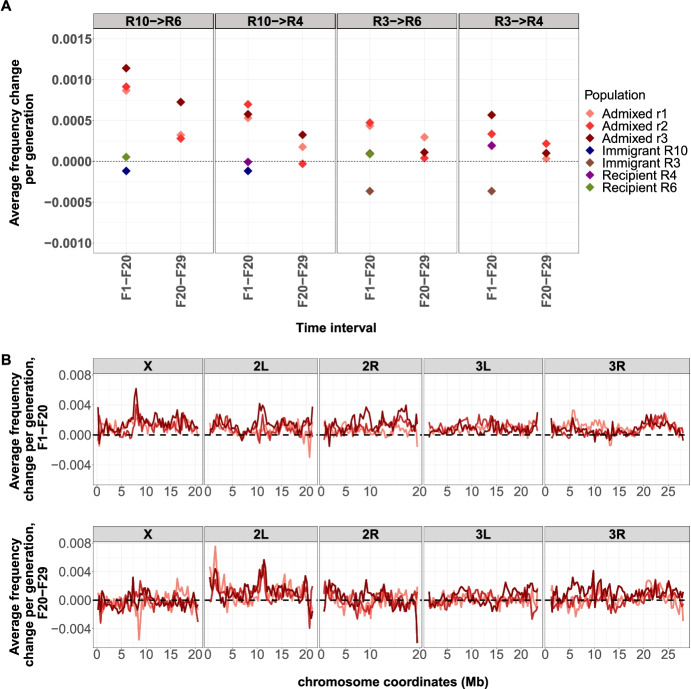
Allele frequency dynamics of alleles selected in the parental immigrant populations after imbalanced mixture. Since the selection targets cannot be mapped, all alleles increasing in frequency during 110 generations in the parental immigrant population are considered as beneficial alleles. **A** In the three replicates the frequency increase is stronger in highly differentiated pairs of evolved founder populations. The allele frequency change in the first period (up to generation 20, dark red) is stronger than in the second phase (generation 21-29, light red). In the founder populations no similar allele frequency change occurs during the same time. **B** This allele frequency increase occurs throughout the entire genome and is stronger in the early phase (up to generation 20, upper panel) than in the later generations (generation 21-29, lower panel)

**Fig. 4:**
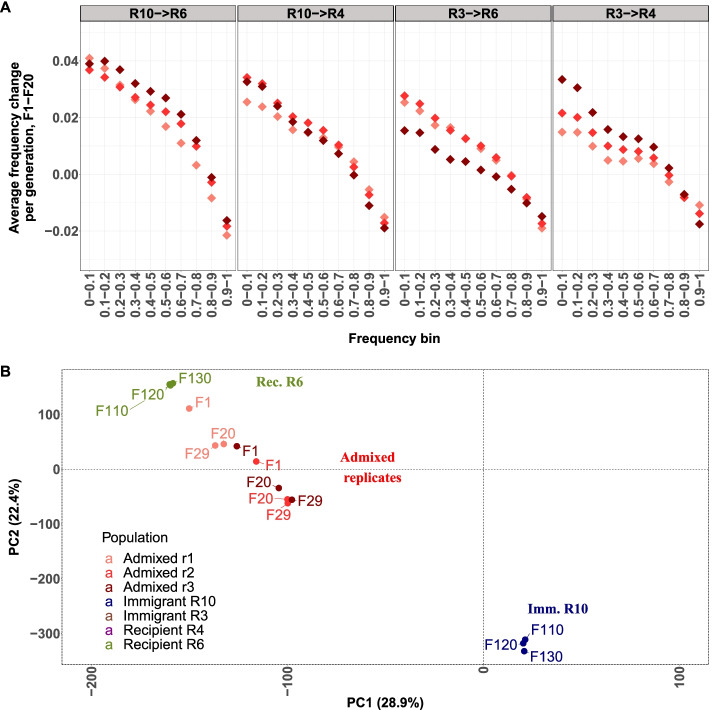
Allele frequency dynamics of alleles selected in the parental immigrant populations after imbalanced mixture (cont.) **A** strong negative correlation between the allele frequency change during the first 20 generations and the starting frequency of the beneficial alleles from the immigrant population in the freshly mixed population. Alleles with a high starting frequency are counter-selected, while low-frequency ones are favored. **B** PCA of the founder populations (generation 110-130) and the mixture of R10 (immigrant population) and R6 (recipient population). In all three replicates the advanced population samples are moving towards the low-frequency founder population-reflecting the non-random allele frequency changes. Each replicate is indicated by a different red color. For clarity the founder populations R3and R4 are not shown. The corresponding plots for other population pairs are given in Suppl. Fig. 4

We tested these predictions experimentally and mixed two evolved populations in unequal proportions (0.15:0.85) and exposed three replicates of four different pairwise combinations for 29 generations in the same hot environment as the evolved founder populations. Since the selection targets are not known, we considered all alleles increasing in the parental population as potentially beneficial alleles. In all mixed populations the beneficial alleles of the immigrant population increased in frequency (Fig. 3A) while the beneficial alleles of the recipient population decreased (Table S3, Fig. S2). The extent to which this pattern can be seen differed between population pairs. Consistent with prediction 2, the frequency increase was more pronounced for highly differentiated populations (Fig. 3A). This pattern cannot be attributed to few genomic regions because the directional frequency increase is seen across the entire genome with considerable heterogeneity across replicates (Fig. 3B). We attribute this heterogeneity among replicates to the combination of stochastic effects at the start of the experiment with a highly polygenic architecture and a large number of associated loci contributing to the pleiotropic cost. The frequency change of alleles beneficial in the immigrant population was highly frequency dependent in the mixed population. Low frequency alleles, which are better masked by heterozygosity, experienced a stronger selection (Fig. 4A). High frequency alleles were counter-selected, which can be explained by the fact that these alleles are selected in both replicates (Fig S3), which does not allow for masking of the pleiotropic costs. Rather, the increase of alleles with masked pleiotropic effects requires frequency change of the other alleles such that the population stays at the trait optimum. For a discussion about alternative interpretation of these results (e.g. deleterious alleles, epistasis, linkage), please see supplementary text.

Interestingly, over the last nine generations, these patterns were less pronounced, which cannot be explained by a less efficient masking due to the frequency increase of these alleles. The observed frequency increase of the low frequency pleiotropic alleles is too small for this. We visualized the directional allele frequency changes in the admixed populations by PCA. As expected by the imbalanced proportions all mixed replicates are in close proximity to one founder population, but generation 20 and 29 show a pronounced movement towards the immigrant population. This pattern is seen most clearly for highly differentiated population pairs, but also holds for weakly differentiated population pairs - although to a lesser degree (Fig. 4B, Fig S4). The large variation during the setup of the mixed replicate populations, most likely due to stochastic sampling of individuals used for the setup of the mixed replicate is reflected in the different position of the F1 generations in the PCA.

**Fig. 5:**
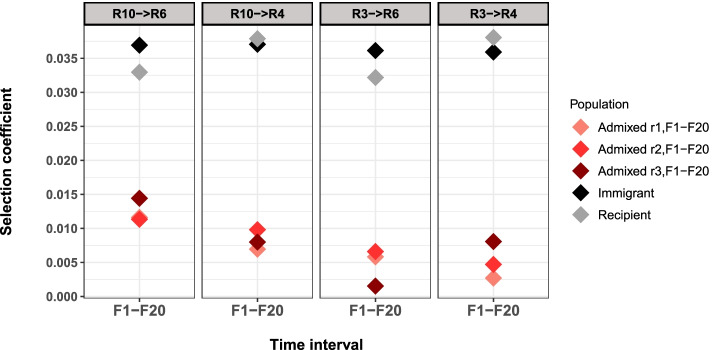
Comparison of the realized selection coefficient to the estimated cost of pleiotropy (shown as the inferred selection coefficient from the admixture experiment F1-F20). Note that the cost of pleiotropy has a positive sign, as we measured it by means of fitness advantage in a mixed population

We estimated the cost of pleiotropy by comparing the mean selection coefficient of beneficial alleles of the recipient population with the selection coefficient of the same alleles in the pure population. Although different between admixture setups, the cost of pleiotropy was substantially lower than the net selection advantage of the beneficial alleles (Fig. 5). This observation is consistent with the lack of significant fecundity differences between freshly mixed populations and mixed populations evolving for 29 generations (Fig. S5). Bearing in mind that our experimental design provides a lower bound for the cost of pleiotropy, we conclude that the pleiotropic costs are low, but still large enough to generate a significant signal in all mixed populations.

## Discussion

Using a novel experimental design specifically tailored to detect pleiotropic costs, we showed that polygenic adaptation to a novel hot temperature environment comes with a small, but detectable cost of pleiotropy. After an imbalanced mixture of two evolved replicate populations, the frequency change of the alleles beneficial in the founder populations was strikingly asymmetric. The beneficial alleles of the immigrant population increased, while those of the recipient population decreased. We propose that this discrepancy reflects the masking of the pleiotropic effects in heterozygous individuals. The beneficial alleles from the immigrant population are less frequent and hence more likely to be heterozygous. Their pleiotropic effects are better masked by the other allele and the observed changes mostly reflect the altered pleiotropic costs. A similar case of dominance heterosis was described for Neanderthal alleles, which increased in frequency despite being deleterious [12]. This interesting dynamic is also reflected by the strong negative correlation between frequency and selective response: alleles beneficial in the immigrant population were more strongly selected in the mixed population when their frequency was low (Fig 3C).

Even the most responsive class of alleles did not change by more than 0.04 after 20 generations, which is not sufficient to explain the decrease in allele frequency change between generation 20 and 29 by a less efficient masking. Rather, we propose that the slower frequency increase of alleles with masked pleiotropic cost can be explained by the mixture dynamics of the two populations. During the first generations only a moderate number of recombination events occurred, which resulted in individuals with rather heterogeneous fitness effects – the number of alleles with masked pleiotropic cost differs among individuals. With an increasing number of generations, these masked alleles are distributed more homogeneously, which in turn reduces the fitness differences among individuals and therefore the frequency increase of alleles with masked pleiotropic cost.

Because immigrant and recipient populations were probably already close to the trait optimum, mixing of two different populations does not result in a shift in trait optimum for codominant alleles. Recessive or dominant alleles will push the mixed population away from the trait optimum, but all alleles will respond in the same direction (suppl. Table 1) and alleles closer to a frequency of 0.5 (i.e. recipient alleles) will exhibit a stronger allele frequency change. Contrary to this, we observed that low frequency alleles beneficial in the immigrant populations displayed the strongest allele frequency change-indicating the impact of pleiotropy. In any case it is highly likely that the dominance differs between both types of effects. While deleterious pleiotropic effects are probably recessive, based on theory [13] and empirical results [14], beneficial mutations are typically (partially) dominant. Consistent with a partially dominant beneficial effect, most alleles favored in the parental replicates started from a low frequency, but reached a high frequency very quickly [11]. Full dominance of the beneficial effect is also unlikely, because we did not observe a fitness reduction after mixing the populations by overshooting the trait optimum (Suppl. Table 1).

Our analyses indicate low, but noticeable costs of pleiotropy. This is in contrast to a model with high (universal) pleiotropy, which predicts that only small effect mutations could contribute to adaptation – at the very extreme pleiotropy may even prevent adaptation [9]. The pronounced allele frequency changes that were seen during 60 generations of adaptation to the novel hot environment [11] clearly speak against pleiotropy restricting adaptation.

Our results seem compatible with previous results of a L-shaped distribution of pleiotropy [5–7], which also imply a low cost of pleiotropy. Nevertheless, we find that the signature of pleiotropic costs is distributed across the entire genome (Fig 3B), which makes it unlikely that a small number of loci with large pleiotropic effects are determining most of the pleiotropic costs. One possible explanation for the lack of loci with strong pleiotropic effects is that their effect sizes are large [5–7] and they are selected in all (most) replicates. Because our approach requires selected loci to occur at different frequency in the replicates, strongly selected loci with a consistent selection response across replicates cannot be identified. Another explanation for the absence of highly pleiotropic loci in our study is that their costs were too high to contribute to adaptation [9].

While we cannot exclude the presence of highly pleiotropic alleles, the genome-wide response in mixed populations resembles polygenic selection responses, in particular in the light of the heterogeneous genomic signatures across the replicate populations. Hence, we propose that our data suggest widespread pleiotropy, but with low costs of pleiotropy. It has been proposed that the cost of pleiotropy is reduced by modularity when mutations affect only a subset of the genes [15]. For example, the analysis of different polymorphisms in the gene *Catsup*, were associated with variation of different phenotypes [16]. Probably a common form of modularity is related to the regulation of gene expression, which restricts transcription to specific tissues, developmental states and physiological conditions [17].

Experimental evolution provided not only strong support for the importance of gene expression changes for adaptation [18–20], but even demonstrated that pleiotropic constraints can be overcome by differential gene regulation. The dopamine pathway is a classic example for pleiotropy, as it affects many phenotypes with neuronal signaling and pigmentation being the most prominent ones [21]. In response to new ambient temperatures neuronal signaling evolved by altering gene expression of key genes in the dopamine signaling pathway, but pigmentation was not affected [22]. Sex-specific adaptive responses are another example of how pleiotropic effects can be avoided, because an evolutionary response is only possible if the sexual conflict (one form of pleiotropy) is avoided/reduced. Sex-specific gene expression changes occurred after 100 generations in a novel environment and were driven by sex-specific transcription factors that enable expression changes in one sex only, while to other sex remains unaffected [23].

This study provided, to our knowledge, the first empirical quantification of pleiotropic costs associated with polygenic adaptation. Consistent with the model of universal pleiotropy, the contributing loci are distributed across the entire genome. Nevertheless, we also noticed that the costs of pleiotropy were much lower than the selective advantage provided by the alleles during adaptation to a new trait optimum. We propose that modularity has reduced the costs of pleiotropy, which allowed a rapid adaptation to a new trait optimum which involved large allele frequency changes. This explains why rapid adaptation even with large allele frequency changes is possible even for polygenic traits.

## Conclusions

We show that allele frequency changes triggered by adaptation to a new environment are accompanied by low, but detectable cost of pleiotropy. Interestingly, many loci with small effect contribute to the cost of pleiotropy.

## Methods

### Experimental design of admixture

We used replicate *D. simulans* populations, which evolved for 110 generations in a novel hot environment fluctuating between 18 and 28°C [11]. Out of the 10 available replicates, we picked two pairs of highly and lowly differentiated populations (Table 1). Two evolved replicate populations were mixed targeting a ratio of 15:85 with a population size of 1250 flies. For every pair of populations, three replicates were generated. The mixed populations were maintained under the same environmental conditions as in [11] for 29 generations.

### Genome sequencing, mapping of sequence reads & SNP calling

We used Pool-Seq [24] to determine genome-wide allele frequencies. In total three time points were sequenced, F1, F20 and F29. Genomic DNA was extracted for all mixed populations with balanced sex ratio. DNA-sequencing libraries were prepared as described in [11] and 100bp paired-end reads resulted in a genome-wide average coverage of about 50x. Reads were trimmed using ReadTools version (1.0.0) to remove the low quality bases (parameters: --mottQualityThreshold 20 --minReadLength 50 --disable5pTrim true). The trimmed reads were mapped using novoalign ( --mapper-args "-i 400,100 -F STDFQ -o SAM -r RANDOM") to the *D. simulans* reference genome [25] on a Hadoop cluster with Distmap version 2.7.5 [26]. Reads in the bam files were sorted and duplicates were removed with Picard version 2.8.1 (http:// broadinstitute.github.io/picard). Reads with low mapping quality and improper pairing were removed (parameters: -q 30 -f 0x0002 -F 0x0004 -F 0x0008) and the bam files were converted to mpileup files using SAMtools version (1.3.1) [27]. The mpileup files were converted to a synchronized pileup file using PoPoolation2 (parameter: --min-qual 20) [28]. Then, repeats (identified by RepeatMasker) and 5-bp regions flanking indels (identified by PoPoolation2: identify-genomic-indel-regions.pl --indel-window 5 --min-count 5) were masked. After these filtering steps, polymorphic positions with a minimum count of 5 were kept. We used SNPs called in [11], but conditioned on the presence at generation 110 and a frequency <0.9. Alleles were polarized to increase in frequency in the parental replicate during 110 generations to determine the beneficial alleles in each of the replicate founder populations.

The selection coefficients (*s*_*i*_) for each locus i were computed as:

$${s}_i=\frac{2}{\varDelta t}\ln \kern0.5em \left(\frac{q_i(f)\left(1-{q}_i(b)\right)}{q_i(b)\left(1-{q}_i(f)\right)}\right)$$

where qi(b) and qi(f) are the starting and end frequencies of a SNP i and Δ*t* is the number of generations. The cost of pleiotropy was estimated by the mean selective advantage of the beneficial alleles from the recipient population in mixed populations. The selection advantage arises from the masking of the pleiotropic effects because they are at low frequency.

### Fecundity

We complemented the fecundity data from generation 103 [11] with measurements at generation 138. After 2 generations of density control at the target temperature regime, we followed the egg counting protocol of [29]. 70 females and 30 males were placed into egg-laying cages and transferred every 12 hours. The eggs on the plate were counted as described in [29]. After 8 transfers males and females were separated, dried, and weighed. For each replicate, we measured fecundity in three replicates.

## Supplementary Information

**Additional file 1:** Supplementary text.

**Additional file 2:** Review history.

## Data Availability

The sequencing data is available in the European Nucleotide Archive (ENA) under the study with accession number PRJEB50633 [30]. All the scripts used for the data analysis and simulations, and the final files (sync files, computed allele frequency files and the fecundity dataset) used for the results presented in the article are available in Zenodo at https://zenodo.org/record/5946858#.Yf1Bl5HMJF [31]. Additional tables and figures underlying the article are available in the supplementary material.
