# Supplementary material for: Natural variation in Drosophila shows weak pleiotropic effects
Source: Genome Biol. 2022 May 16;23:116. doi: 10.1186/s13059-022-02680-4 (PMC9109288; doi:10.1186/s13059-022-02680-4)
Supplement: Supplementary file 1 — Additional file 1: Supplementary text. [file 13059_2022_2680_MOESM1_ESM.docx]

**Title:** Low cost of pleiotropy during polygenic adaptation to a novel environment

Supplementary Material

[Supplementary text 2](#_Toc94434946)

[Confirmation of the expected effect of pleiotropy on the allele frequency changes in admixed populations 2](#_Toc94434947)

[Computer simulations rule out that the observed patterns of allele frequency change can be obtained under neutrality 2](#_Toc94434948)

[Simplifying assumptions 2](#_Toc94434949)

[Linkage 3](#_Toc94434950)

[Recessive deleterious alleles 3](#_Toc94434951)

[Inbreeding 3](#_Toc94434952)

[Epistasis 4](#_Toc94434953)

[Supplementary methods: computer simulations 5](#_Toc94434954)

[Computer simulations to demonstrate the impact of pleiotropy on allele frequency changes after mixture of two evolved populations 5](#_Toc94434955)

[Neutral computer simulations 6](#_Toc94434956)

[Simulating epistasis 6](#_Toc94434957)

[Supplementary Tables 8](#_Toc94434958)

[Supplementary Figures 12](#_Toc94434959)

# Supplementary text

## Confirmation of the expected effect of pleiotropy on the allele frequency changes in admixed populations

The patterns of allele frequency change in the simulations (see suppl. methods) nicely match the empirical data. The rising alleles in the immigrant populations increased their frequency in the admixed populations while we observed nearly no allele frequency changes in the parental immigrant/recipient populations between F101 and F120 (Suppl. Figure 1a). We also observed that the beneficial alleles from the immigrant population at low frequency in the admixed population were favored (Suppl. Figure 1b) while the beneficial alleles from the recipient population were counter-selected (Suppl. Figure 1c), which has also been seen in the empirical results. PCA plots (Suppl. Figure 1d) also showed that the admixed population is moving towards the immigrant population. These results were consistent with the observation in the empirical data, suggesting that recessive pleiotropic cost can explain the observed patterns in allele frequency changes after admixture.

## Computer simulations rule out that the observed patterns of allele frequency change can be obtained under neutrality

We simulated neutral evolution after the admixture event and unlike the empirical data we did not observe allele frequency changes after the admixture event (Suppl. Fig. 7A). Based on these simulations we conclude that genetic drift alone cannot explain the empirical results.

## Simplifying assumptions

Our analyses made some simplifying assumptions and did not explicitly account for important population genetic parameters. Hence, we discuss the potential consequences of this on the interpretation of our observations, but conclude that these factors are unlikely to result in the observed patters.

### Linkage

Like other studies of polygenic adaptation [1], we did not focus on few significant loci, but rather combine the signal across many loci. We consider each SNP, which is increasing in one of the founder populations as a target of selection. This bears the caveat that SNPs linked to a target of selection will also be considered as selection target. The impact of linkage depends on the underlying genetic architecture, when only a small number of selection targets are driving adaptation low frequency selection targets will result in long selected haplotype blocks, while for those with higher starting frequency the selected haplotype block will be shorter [2]. This implies that the weight of loci depends on their frequency, potentially distorting the result. At the infinitesimal limit, each haplotype block reflects the net effect of all loci in a selected haplotype block, which smoothens the estimated effects without biasing the mean. Since the adaptive architecture of temperature adaptation is highly polygenic, our results should not be strongly biased by linkage.

### Recessive deleterious alleles

Another implication of linkage is that we cannot distinguish between pleiotropic costs of a selected allele and unconditionally deleterious allele linked to a locus contributing to the trait. Fully recessive unconditionally deleterious alleles will show the same dynamics as adaptive alleles with pleiotropic effects. Since dominance coefficients as low as 0.1 already result in counter selection [3], they are unlikely to increase in frequency. The frequency of recessive deleterious alleles are not sufficiently well known to estimate their influence in our study. One particularly strong argument against unconditionally deleterious alleles driving this pattern comes from the behavior of X-linked loci. Hemizygous X-chromosomes in males facilitate the purging of recessive deleterious alleles on the X chromosome, but we observed the same allele frequency dynamics for X-linked loci (Suppl. Fig. 6). Even if recessive deleterious alleles also contribute to the signal, which we attribute to pleiotropic costs, this implies that pleiotropic effects are even weaker than estimated – hence recessive deleterious alleles make our test conservative.

### Inbreeding

Because we mixed two populations, which have been maintained at moderate population sizes, it may be possible that the increase of the low frequency beneficial alleles reflects amelioration of inbreeding depression. We consider this an unlikely explanation because even after more than 100 generations in the laboratory the parental populations maintained considerable molecular variation (see Supplementary Table 4). Furthermore, we found no fitness differences between parental and F1 lines (Supplementary Fig. 4) and the parental populations have very few (<66) private alleles (with a frequency of less than <0.05 in the other population), which could compensate for fixation of deleterious alleles in one of the parental populations.

### Epistasis

Mixing diverged populations could also change the epistatic interactions that were established during more than 100 generations of adaptation. While epistatic effects have been described to be abundant and strong [4-6], we do not expect a directional frequency increase of the beneficial alleles in the recipient population. Rather epistasis could either increase, decrease or reverse the effects, thus the frequency should change in both directions. We scrutinized these expectations with forward computer simulations. As expected, we did not find a pronounced allele frequency change caused by pleiotropic effects (Supplementary Figure 7b). Since we see the strong directional bias in allele frequency change in the empirical data, we conclude that epistasis is unlikely to be the driving force.

# Supplementary methods: computer simulations

## Computer simulations to demonstrate the impact of pleiotropy on allele frequency changes after mixture of two evolved populations

We performed computer simulations to support our hypothesis that pleiotropic effects of alleles favored during the first phase provide a fitness advantage after mixing evolved populations in unequal proportions. Simulating pleiotropy without further information about essential parameters, such as the number of affected traits, correlation between traits and the genetic architecture of the traits affected by pleiotropic effects is a daunting task. Hence, we decided to simplify the simulations by assuming directional selection, where the selection coefficient reflects the combined effects of positive selection and pleiotropy. We assumed two different phases – before (phase 1) and after admixture (phase 2). Simulations were performed with MimicrEE2 [7].

In the first phase, two replicate populations evolved under the same selection regime from the same founder population. Selection started from 300 diploid individuals (the effective population size of the experimental populations is ~300 [8]). Each diploid individual was reconstructed by randomly combining 2 out of 189 phased ancestral *Drosophila simulans* haplotypes [8] and assuring that each haplotype has the same probability to be chosen. From all segregating sites in this founder population, we randomly sampled 100 selected loci. Recombination rates were taken from [9]. For each of the 100 selected loci, we first assigned fitness 1.5, 1.25 and 1 to genotypes AA, Aa and aa to represent the selective advantage. To account for the recessive pleiotropic cost of each locus, we add additional negative effects to the fitness of genotypes. The negative effects were -0.25, -0.025 and 0 for genotypes AA, Aa and aa correspondingly. The final fitness for each locus provided in the simulations for each genotype was 1.25 (AA), 1.225 (Aa) and 1 (aa). The two populations with the same genetic architecture independently evolved for 100 generations. After 100 generations of evolution for both populations, we mixed 15% of the individuals from the population 1 (defined as immigrant population) and 85% of the individuals from the population 2 (defined as recipient population) to create the admixed population.

In the second phase, where the admixed population is at generation 1 and the immigrant/recipient population are at generation 100, we reassigned the selection advantage for each locus in three populations (immigrant, recipient and admixed populations) assuming the populations have approached the trait optimum such that the selection advantage has decreased but the cost remains constant. For each of the 100 selected loci, the selection advantage was reassigned to 1.4, 1.2 and 1 for genotypes AA, Aa and aa, but the cost remained the same (-0.25, -0.025 and 0 for genotypes AA, Aa and aa). The final fitness for each genotype was 1.15 (AA), 1.175 (Aa) and 1 (aa), which represent the reduced selection, in phase 2 for all three populations. The immigrant (start from F100), recipient (start from F100) and admixed populations (start from F1) with 300 diploid individuals evolved for 20 generations independently under the re-defined fitness parameters (immigrant population: F101-F120; recipient population: F101-F120; admixed population: F1-F20). The allele frequencies of the simulated loci were recorded every 5 generations. We estimated the heterogeneity of these simulations by repeating phase 1 and phase 2 for 100 times.

The analysis of the simulated data followed the same protocol as the empirical data.

## Neutral computer simulations

To ensure that the frequency changes after admixture are not a result of genetic drift, we simulated neutral evolution for 30 generations in a population with 300 diploid individuals (the effective population size of the experimental populations is ~300 [8]). Because the linkage structure after admixture is not known, we accounted for linkage by using 189 *D. simulans* haplotypes [8], as described above. While this strategy does not account for recombination and drift during the 100 generations of the first phase, we still considered it the best guess for the linkage structure after admixture. For the simulations we sampled 500,000 SNPs from chromosomes 2 and 3 of the 300 diploid individuals matching the frequency distribution of SNPs in the admixed populations. Recombination rates of *D. simulans* were taken from [9]. 100 replicates of neutral evolution were simulated with the function w of MimicrEE2 [7] (version v206). Allele frequencies were recorded every 10 generations.

## Simulating epistasis

We used computer simulations to explore the impact of epistasis on the allele frequency changes after admixture. Similar to the neutral simulations we sampled 500 loci from 189 phased ancestral *Drosophila simulans* haplotypes [8] matching the allele frequency distribution in the admixed populations. For 125 pairs of loci we assumed positive epistatic interactions and 125 pairs of loci had negative epistatic interactions. We assigned the fitness of 1 to genotypes Ab, ab and aB and fitness 1.01 to genotype AB. Similarly, to model negative epistasis we assigned fitness 1 to genotypes Ab, ab and aB and fitness 0.99 to genotype AB. 100 replicates of a population of 300 haploid individuals were simulated with MimicrEE2 [7] (version v206) for 30 generations using the recombination map of *D. simulans* [9].

# Supplementary Tables

**Table S1:** Impact of the mode of inheritance on the expected allele frequency change of alleles beneficial in the immigrant and recipient populations

| Pleiotropic effects | Focal effects | Position relative to trait optimum | Allele frequency change of recipient allele | Allele frequency change of immigrant allele |
| --- | --- | --- | --- | --- |
| Dominant | Dominant | Right^1^ | - | - |
| Dominant | Recessive | Left^2^ | + | + |
| Dominant | Codominant | At optimum | o | o |
| Recessive | Dominant | Right^1^ | -- | +/o/-^5^ |
| Recessive | Recessive | Left^2^ | + | ++^3^ |
| Recessive | Codominant | At optimum | -^4^ | +^4^ |
| - | Dominant | Right^1^ | - | - |
| - | Recessive | Left^2^ | + | + |
| - | Codominant | At optimum | o | o |

-: frequency decrease, +: frequency increase, o: no frequency change

1: corresponds to overshooting, 2: corresponds to a lower trait value 3: stronger allele frequency change due to the synergistic action of selection towards trait optimum and lower cost of pleiotropy 4: alleles move in opposite direction to assure maintenance of trait optimum, 5: depending on the extent of overshooting relative to pleiotropic effect, but overall a stronger allele frequency decrease is expected for alleles beneficial in the recipient population.

While the magnitude of the effects depend on many unknown parameters (e.g. shape of fitness function for focal trait and fitness function(s) for pleiotropic effects), the best fit to our empirical data is expected for recessive pleiotropic effects and codominant or dominant focal effects.

**Table S2:** Toy calculation explaining how an imbalanced mixture changes the proportion of heterozygous alleles, which in turn causes differential selection responses for partially recessive alleles

| Origin of beneficial allele | Allele frequency | Genotype frequency: p^2^ | Genotype frequency:  2pq | 2pq/p^2^ |
| --- | --- | --- | --- | --- |
| low frequency population | 0.6*0.15=0.09 | 0.0081 | 0.1638 | 20.2 |
| high frequency population | 0.6*0.85=0.51 | 0.2601 | 0.4998 | 1.9 |

**Table S3:** Mean frequency change per generation of the beneficial alleles of the immigrant (upper table) and recipient (lower table) population in the admixed replicates between generations 1-20. Standard error is shown in parenthesis.


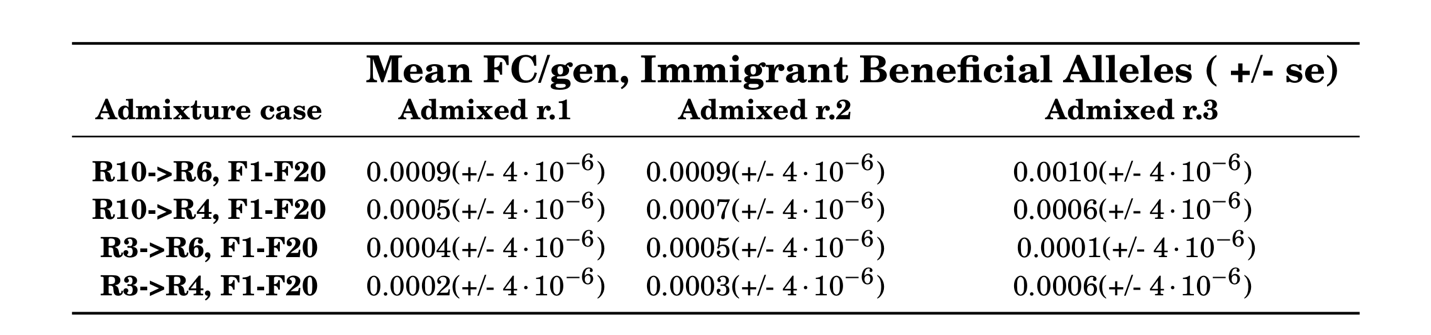


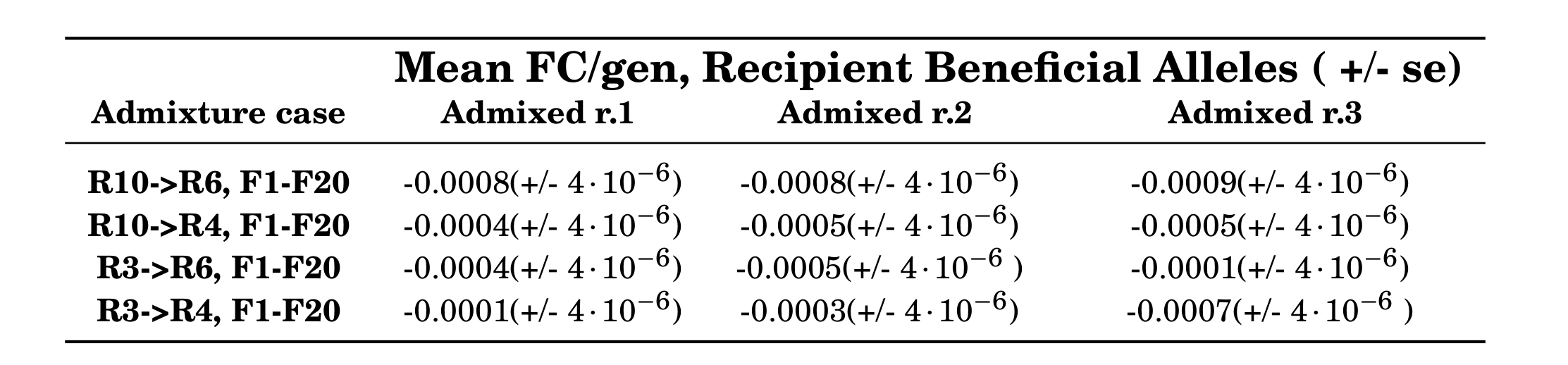


**Table S4:** Variability of the evolved populations is very similar to the ancestral founder populations


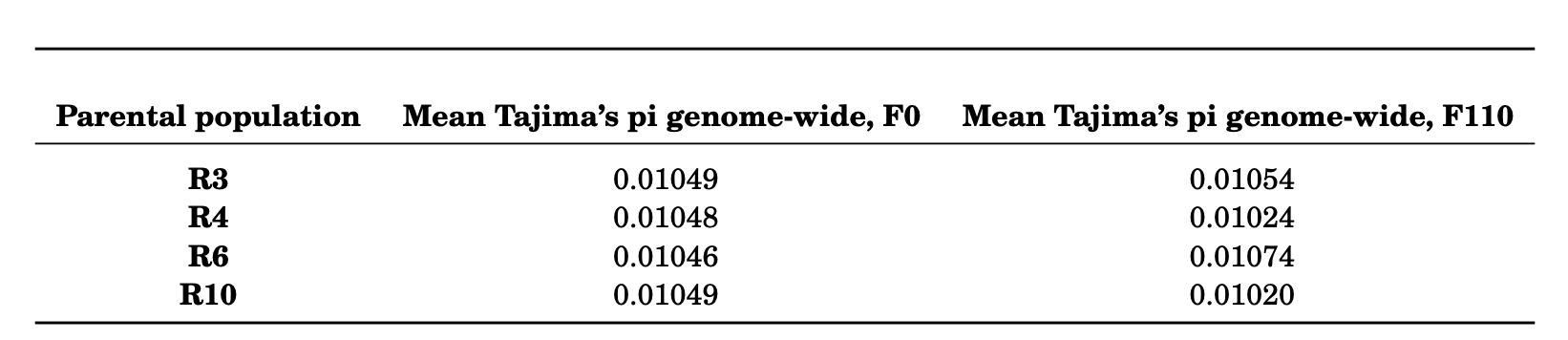


# Supplementary Figures

**Figure S1:** Validation of the allele frequency dynamics after admixture predicted for the presence of pleiotropic costs by computer simulations. A) The alleles rising in frequency (beneficial) during the first phase in the immigrant (Imm.) population increase in frequency after mixture (dark red; F1-F20) while in the parental populations almost no frequency change was observed (grey and dark grey; F101-F120). B) Average frequency change (F1-F20) for the alleles with rising frequencies in the immigrant population is negatively correlated with its starting frequency in the admixed population. The low frequency ones were strongly selected for. C) Alleles increasing in frequency in the recipient (Rec). population from generation 1 to 100 decrease in frequency after mixture. Th mean frequency change per generation of the beneficial alleles of the recipient population in the admixed (F1-20) and parental (F101-120) populations is shown. D) PCA of one simulation round showing the recipient (grey) and immigrant (dark grey) populations (generation 101-120) and the admixed populations (dark red). The admixed population is moving towards the immigrant population-reflecting the non-random allele frequency changes.


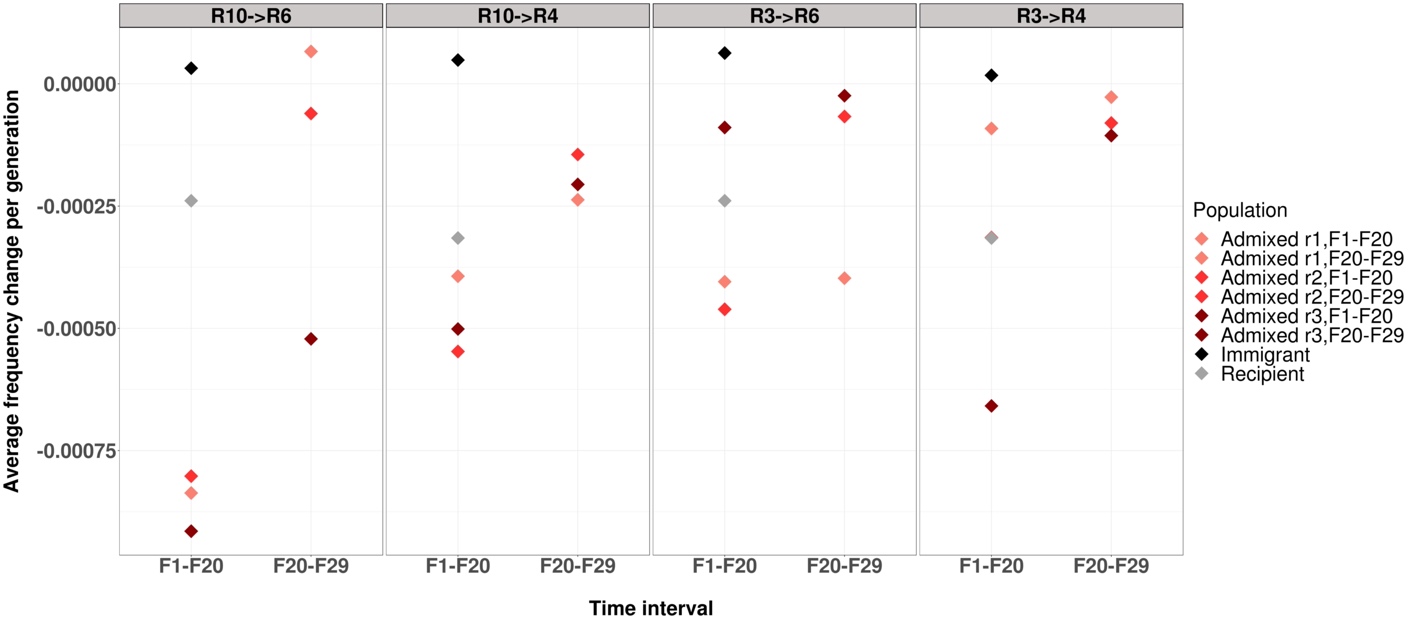


**Figure S2:** The alleles beneficial in the recipient population decrease after mixture. Mean frequency change per generation of the beneficial alleles of the recipient population in the admixed (generation 1-20, 20-29) and parental (110-130) populations is shown.


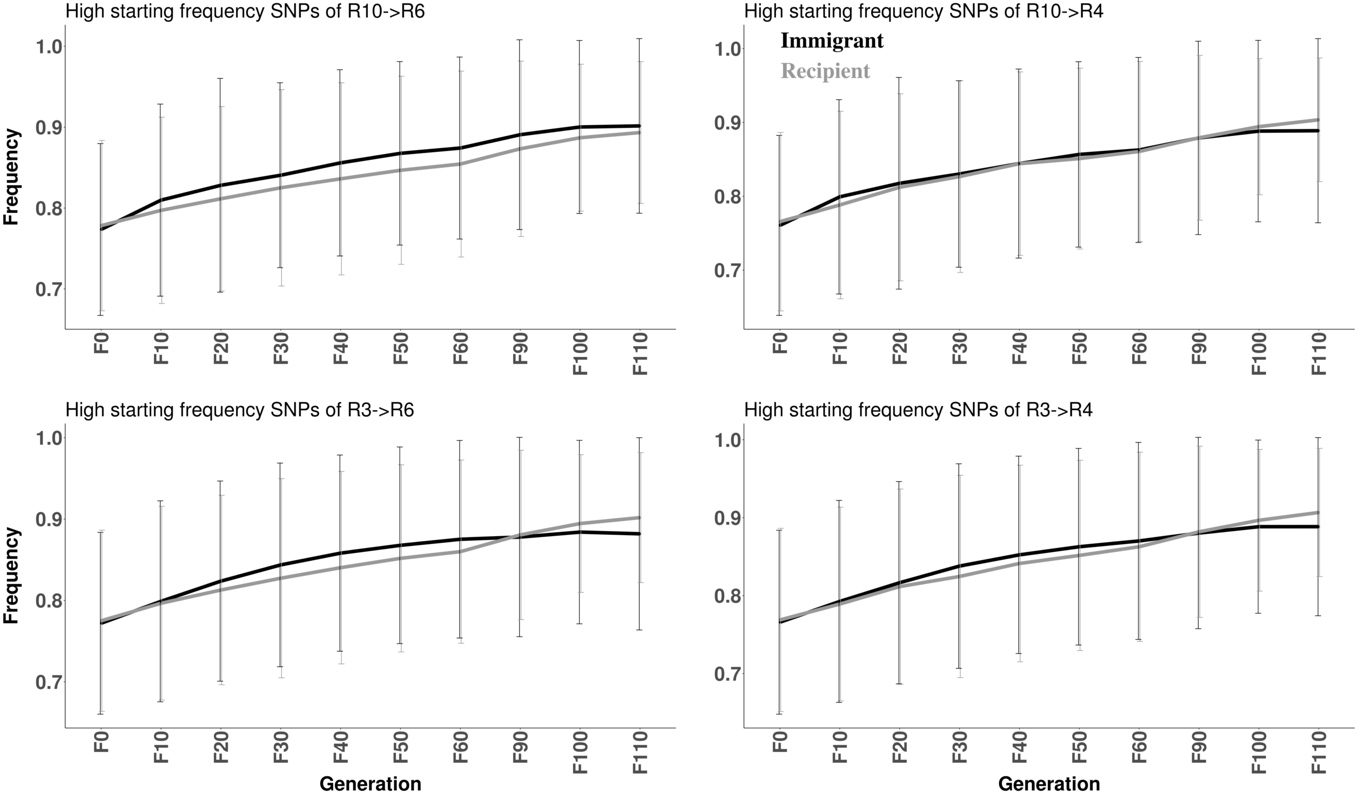


**Figure S3:** Response of the high frequency alleles of generation 1 of the admixed populations in the parental samples. High frequency alleles were counter-selected in the admixed populations because these alleles are selected in both parental replicates as it is shown by the frequency trajectories. (Mean frequency with standard deviation bars is plotted for each generation).


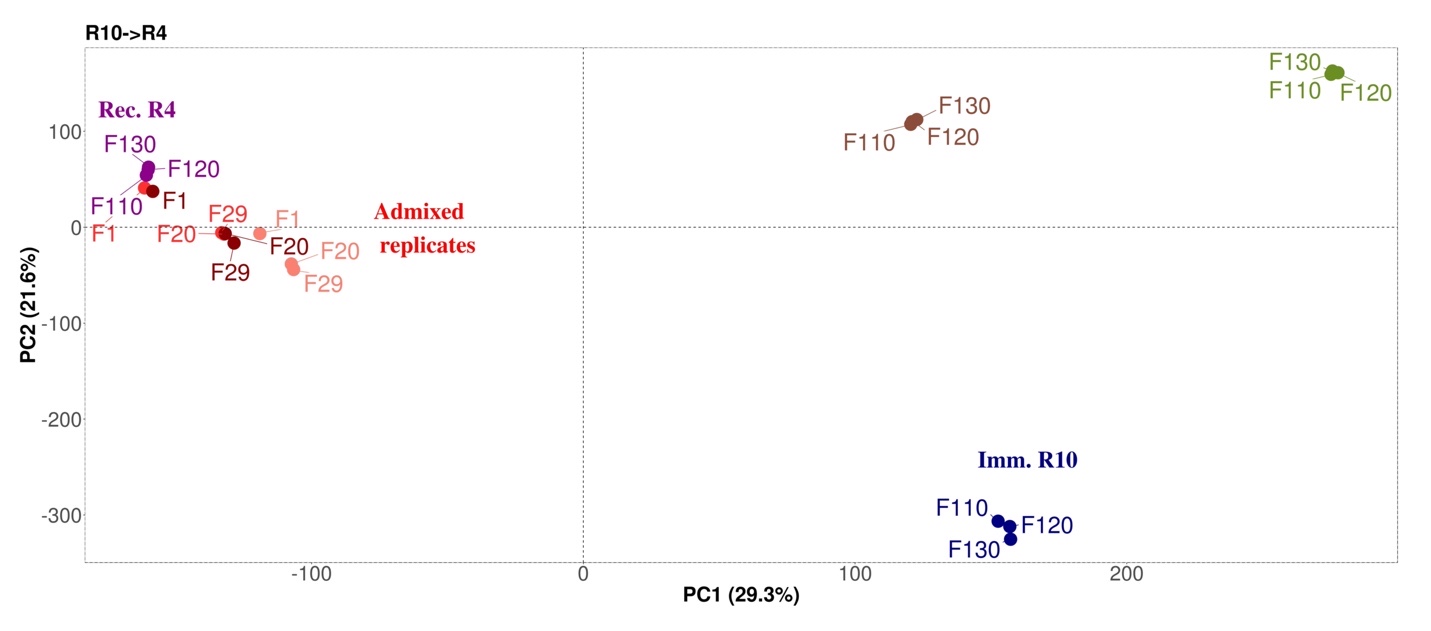

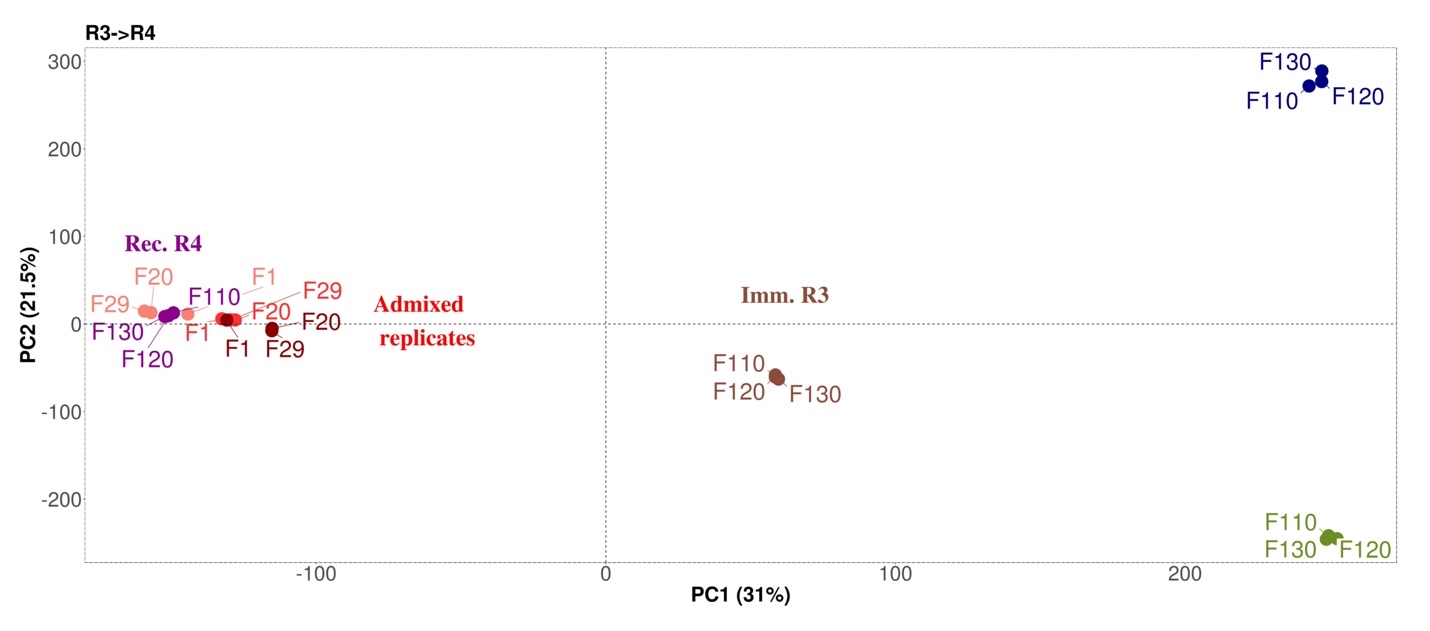

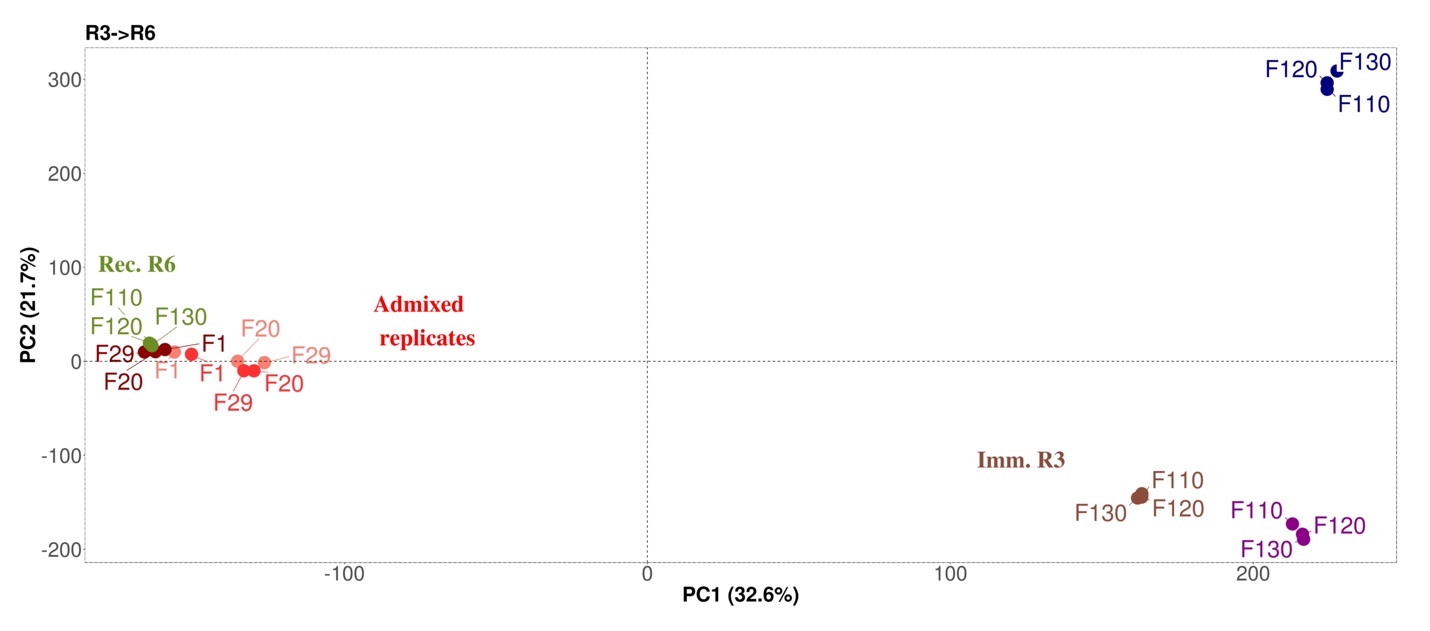


**Figure S4:** Principal Component Analysis (PCA) of the founder populations (generation 110-130) and the mixture of two evolved populations. Three replicates are shown for each admixed population. Each replicate is indicated by a different red color. The header indicates the population pair, with the first population being the immigrant and the second one the recipient one.


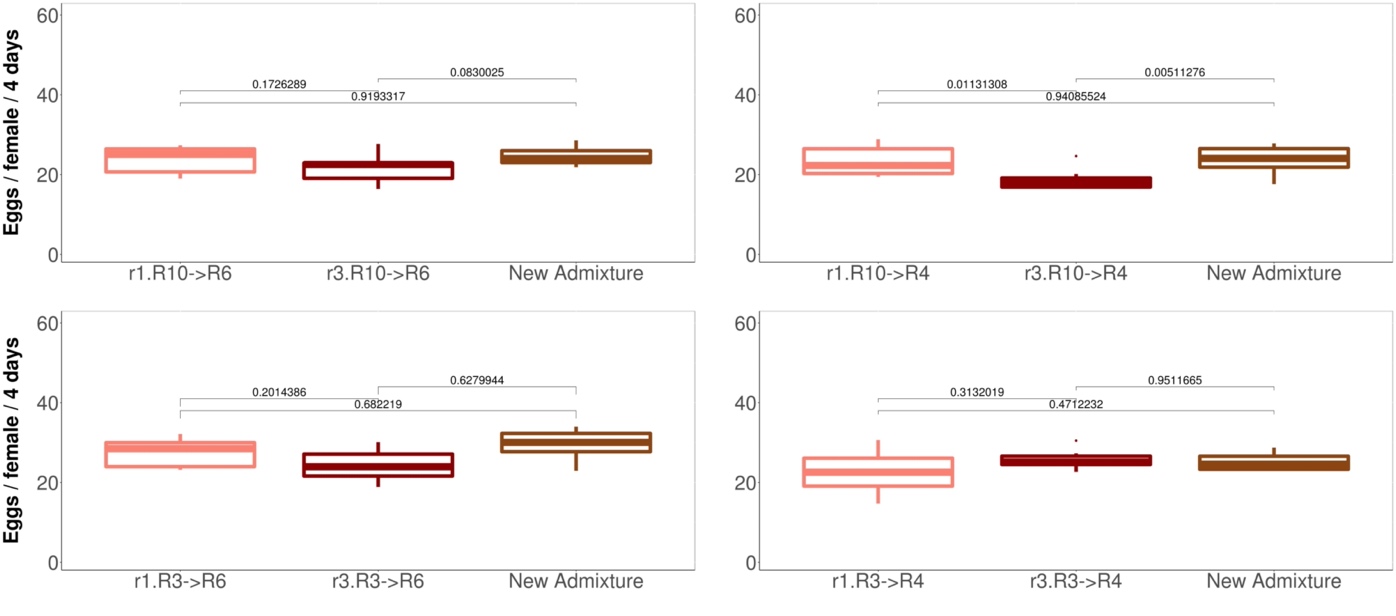


**Figure S5:** Lack of significant fecundity differences between freshly mixed populations and mixed populations evolving for 26 generations.


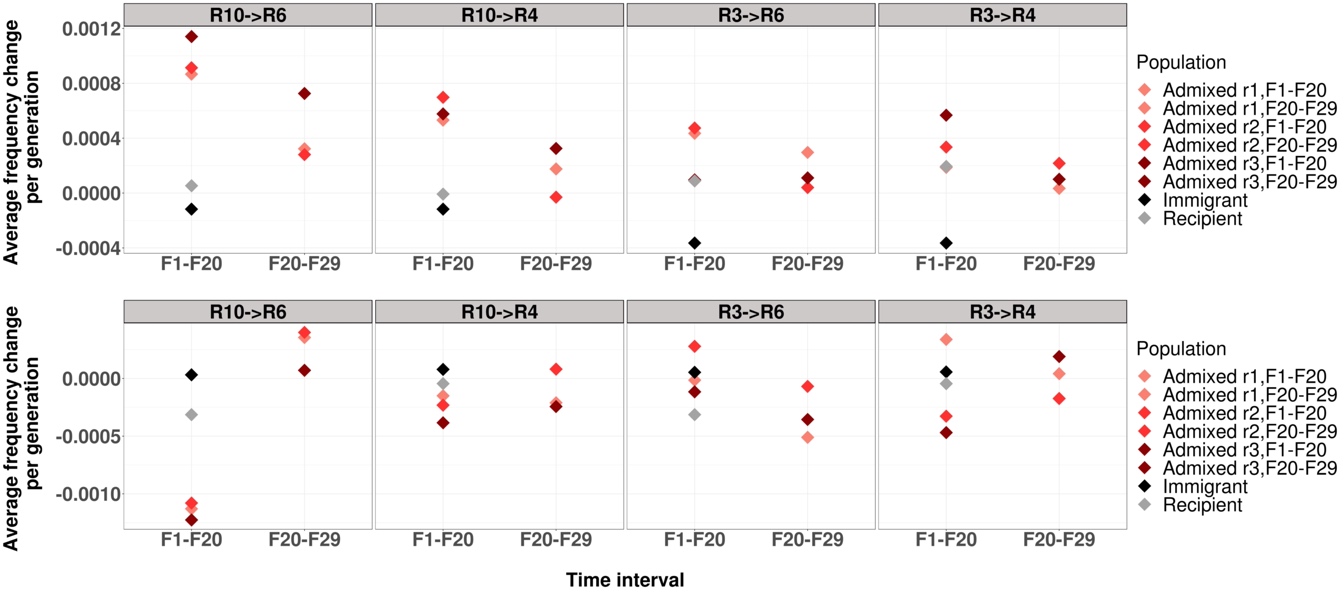


**Figure S6:** Similar allele frequency changes X-chromosomal SNPs suggest that the pattern is not driven by recessive unconditional deleterious alleles. Mean frequency change of the beneficial alleles of the immigrant (upper) and the beneficial alleles of the recipient (lower) population that are located in chromosome X. Their response does not differ from the response of the autosomal SNPs.


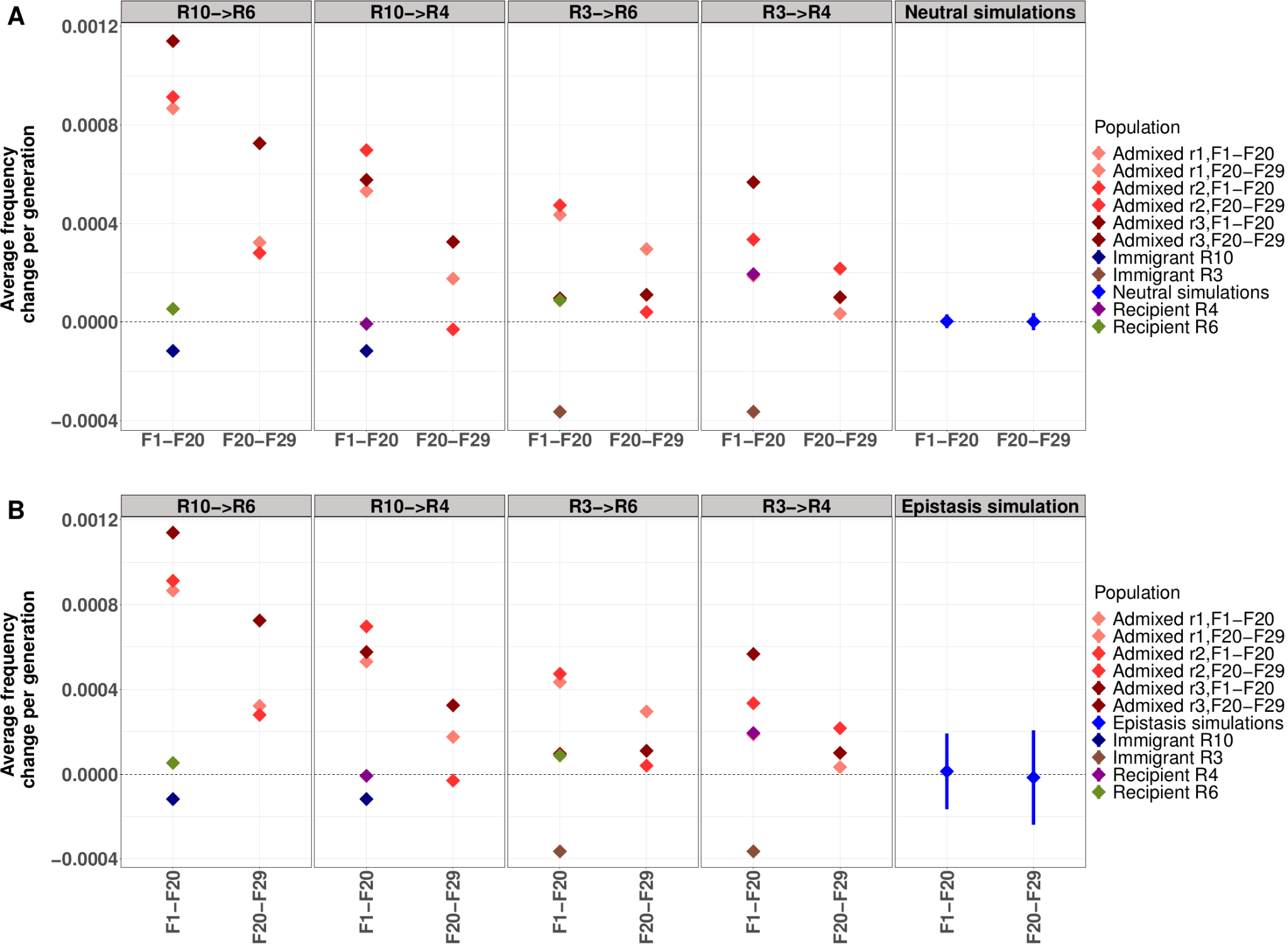


**Figure S7:** Average frequency change per generation in the experimental data vs simulated data in the case of neutral evolution (A) or epistasis (B). The bars in the simulated data show the standard deviation computed from the values of the 100 simulated replicate populations.
