# Supplementary material for: Natural variation in Drosophila shows weak pleiotropic effects
Source: Genome Biol. 2022 May 16;23:116. doi: 10.1186/s13059-022-02680-4 (PMC9109288; doi:10.1186/s13059-022-02680-4)
Supplement: Supplementary file 2 — Additional file 2: Review history. [file 13059_2022_2680_MOESM2_ESM.docx]

**Review history**

**First round of review**

**Reviewer 1**

This is a very interesting paper. The authors designed a clever experiment to test whether adaptive changes are accompanied by pleiotropic costs for polygenic fitness. The experiment takes advantage of a previously warm adapted set of D. simulans populations, where a previous study from the same group showed that different loci contributed to the fitness increase in replicate populations. Therefore when diverged populations are mixed, beneficial alleles at low frequency (thus deleterious effects masked by dominant common allele) will have the opportunity to increase in frequency. The extent of such increase provides an indirect way to measure pleitropic costs. The authors showed, in replicate admixed populations, that this is indeed the case, i.e. adaptively advantageous alleles do come at a pleitropic cost. However, the increase in allele frequency is weak in magnitude. Then the authors went on to propose that this might be due to modularity, which I think is unnecessary. The paper is straightforward and easy to read. The experiment is clever and the authors obviously have thought very deeply about these problems.

I have only one major concern, which is how to distinguish the observed changes from simple genetic drift. The size of the popualation is 1250 but the effective population size could be much smaller. In addition, low frequency alleles increae in frequency and high frequency alleles decrease is completely consistent with drift, simply because the allele freqquencies have to move in some direction and one has a larger space to move than the other. The results are often summarized as averages across the genome, which may mask some interesting patterns. I suggest the authors do a simulation to tease drift and pleiotropy apart. Simply simulate the population mixture with the starting frequencies observed in the ancestral populations and summarize the same statistics. Use these as a background, on which to put the observed results in context.

p3, l57: delete "use"
p9, l55: it looks like each replicate is indicated by a different shape rather than color
p14, l34: please cite the MBE paper that described this estimation approach

**Reviewer 2**

This paper describes a new experimental design and analysis strategy to measure the extent of pleiotropy in polygenic traits. In the experiment, replicate populations are moved to a new environment and evolved for multiple generations. Then admixed populations are created between the replicates and again placed in the new environment for multiple generations. The authors hypothesize that if there is extensive pleiotropy, different sets of alleles will rise to high frequency in the initial selection experiment. Then, during the admixture experiment, the starting allele frequencies, the relative effects of pleiotropy, and fitness will dictate the changes in allele frequency. The authors apply this approach to a fly population and observe changes consistent with their idea. It is a very clever experiment and a largely well written paper. I have the following comments:

My main concern is the lack of theory, empirical simulation, or citation to back up the author's intuition. While many of the claims in the paper feel right and seem correct, it would be very helpful to show that this is the case. This should be quite straightforward in many cases, and could be done via simulation where it is not. Many of the comments below could be addressed by these additions.

In Figure 2B, it looks like the fecundity of R4 is quite a bit lower than R10. Is this difference small enough to ignore? If so, can you explain why? How large a difference would be concerning?

On a related note, if R10 has higher fecundity than R3, as it appears in Figure 2, could this also lead to larger increases observed in Figure 3a? Is there a way to adjust Figures 3 and 4 to account for fecundity differences?

Figure 2A and similar supplementary figures would be clearer with more distinct colors.

Couldn't epistasis (as opposed to pleiotropy) also produce differences in the allele frequencies observed after the first round of selection? In the supplement the authors claim, but do not show, that epistasis will not lead to the observed effect in allele frequency shifts after admixture.

Within the paper, not just Supp Table 1, make clear that changes in frequency are dominance structure dependent. Which phenotypic model is being assumed here? Is there evidence in such widespread dominance (as opposed to additive) effects?

**Authors’ response to reviewers**

Reviewer 1:

This is a very interesting paper. The authors designed a clever experiment to test whether adaptive changes are accompanied by pleiotropic costs for polygenic fitness. The experiment takes advantage of a previously warm adapted set of D. simulans populations, where a previous study from the same group showed that different loci contributed to the fitness increase in replicate populations. Therefore when diverged populations are mixed, beneficial alleles at low frequency (thus deleterious effects masked by dominant common allele) will have the opportunity to increase in frequency. The extent of such increase provides an indirect way to measure pleitropic costs. The authors showed, in replicate admixed populations, that this is indeed the case, i.e. adaptively advantageous alleles do come at a pleitropic cost. However, the increase in allele frequency is weak in magnitude. Then the authors went on to propose that this might be due to modularity, which I think is
unnecessary. The paper is straightforward and easy to read. The experiment is clever and the authors obviously have thought very deeply about these problems.

**We appreciate the very positive feedback of the reviewer about our experiment!**

I have only one major concern, which is how to distinguish the observed changes from simple genetic drift. The size of the popualation is 1250 but the effective population size could be much smaller. In addition, low frequency alleles increae in frequency and high frequency alleles decrease is completely consistent with drift, simply because the allele freqquencies have to move in some direction and one has a larger space to move than the other. The results are often summarized as averages across the genome, which may mask some interesting patterns. I suggest the authors do a simulation to tease drift and pleiotropy apart. Simply simulate the population mixture with the starting frequencies observed in the ancestral populations and summarize the same statistics. Use these as a background, on which to put the observed results in context.

**Following the advice of the reviewer, we have performed neutral computer simulations and show that the pattern from the empirical data cannot be reproduced with neutral simulations. We added this analysis to the supplement. As expected, the pronounced allele frequency change after admixture cannot be explained by genetic drift.**

**Supplementary Figure 7A: Average frequency change per generation in the experimental data vs simulated data in the case of neutral evolution. The bars in the simulated data show the standard deviation computed from the values of the 100 simulated replicate populations.**

p3, l57: delete "use"

**done**

p9, l55: it looks like each replicate is indicated by a different shape rather than color

**we have now changed the color code to make it consistent throughout the manuscript.**

p14, l34: please cite the MBE paper that described this estimation approach

**done**

Reviewer 2:

This paper describes a new experimental design and analysis strategy to measure the extent of pleiotropy in polygenic traits. In the experiment, replicate populations are moved to a new environment and evolved for multiple generations. Then admixed populations are created between the replicates and again placed in the new environment for multiple generations. The authors hypothesize that if there is extensive pleiotropy, different sets of alleles will rise to high frequency in the initial selection experiment. Then, during the admixture experiment, the starting allele frequencies, the relative effects of pleiotropy, and fitness will dictate the changes in allele frequency. The authors apply this approach to a fly population and observe changes consistent with their idea. It is a very clever experiment and a largely well written paper.

**We thank the reviewer for the positive feedback.**

I have the following comments:

My main concern is the lack of theory, empirical simulation, or citation to back up the author's intuition. While many of the claims in the paper feel right and seem correct, it would be very helpful to show that this is the case. This should be quite straightforward in many cases, and could be done via simulation where it is not. Many of the comments below could be addressed by these additions. 

**We agree with the reviewer and have followed the advice and included simulations in the presence of pleiotropic effects. Our results confirm the prediction that alleles from the immigrant population experience a selective advantage after admixture. The results and description of the simulations are provided in the supplement.**

**Supplementary Figure 1: Validation of the allele frequency dynamics after admixture predicted for the presence of pleiotropic costs by computer simulations. A) The alleles rising in frequency (beneficial) during the first phase in the immigrant population increase in frequency after mixture (dark red; F1-F20) while in the parental populations almost no frequency change was observed (grey and dark grey; F101-F120). B) Average frequency change (F1-F20) for the alleles with rising frequencies in the immigrant population is negatively correlated with its starting frequency in the admixed population. The low frequency ones were strongly selected for. C) Alleles increasing in frequency in the recipient population from generation 1 to 100 decrease in frequency after mixture. Th mean frequency change per generation of the beneficial alleles of the recipient population in the admixed (F1-20) and parental (F101-120) populations is shown. D) PCA of one simulation round showing the recipient (grey) and immigrant (dark grey) populations (generation 101-120)**

In Figure 2B, it looks like the fecundity of R4 is quite a bit lower than R10. Is this difference small enough to ignore? If so, can you explain why? How large a difference would be concerning?

**We agree with the reviewer that the fact that the difference in fecundity between the replicate populations is preserved at different time points provides a strong signal of biological effects, rather than stochastic noise. Our explanation is that fecundity does not equate to fitness, but it is only a fitness component. Hence, we reason that overall fitness may be similar among the lines, but fecundity differs. It may be possible that R4 has a higher survival rate or better mating success than R10. Some support for this comes from the comparison of the introgression of R10 in R6 and R4. R6 has a higher fecundity than R4, implying that R10 should experience a smaller advantage in combination with R6 compared to R4. Nevertheless, we observe the opposite pattern. Hence, we think that the difference in fecundity is not the driver of the observed pattern and the difference between lines can be neglected.**

On a related note, if R10 has higher fecundity than R3, as it appears in Figure 2, could this also lead to larger increases observed in Figure 3a? Is there a way to adjust Figures 3 and 4 to account for fecundity differences?

**See above. In addition, we would like to mention that it is not clear whether the fecundity differences persist after mixing of the genotypes after F1.**

Figure 2A and similar supplementary figures would be clearer with more distinct colors.

**We changed the color scheme.**

Couldn't epistasis (as opposed to pleiotropy) also produce differences in the allele frequencies observed after the first round of selection? In the supplement the authors claim, but do not show, that epistasis will not lead to the observed effect in allele frequency shifts after admixture.

**We performed simulations with epistasis and did not find a similar pattern as in the experimental data. We think that the reason for this is that the direction of epistasis is random, hence its effect cancels out. The epistasis simulations were included in the supplement.**

**Supplementary Figure 7B: Average frequency change per generation in the experimental data vs simulated data in the case of epistasis. The bars in the simulated data show the standard deviation computed from the values of the 100 simulated replicate populations.**

Within the paper, not just Supp Table 1, make clear that changes in frequency are dominance structure dependent. Which phenotypic model is being assumed here? Is there evidence in such widespread dominance (as opposed to additive) effects?

**In the introduction we state: “In a mixture of the two populations recessive pleiotropic effects are (partially) masked in heterozygous individuals. If the mixture is imbalanced and pleiotropic effects are not fully dominant, adaptive alleles from the two populations experience different pleiotropic costs due to their frequency in the mixed population (suppl. Table 1, 2).”**

**In the discussion we discuss the issue of dominance for beneficial and deleterious alleles:**

**“While deleterious pleiotropic effects are probably recessive, based on theory [13] and empirical results [14], beneficial mutations are typically (partially) dominant. Consistent with a partially dominant beneficial effect, most alleles favored in the parental replicates started from a low frequency, but reached a high frequency very quickly [11]. Full dominance of the beneficial effect is also unlikely, because we did not observe a fitness reduction after mixing the populations by overshooting the trait optimum (Suppl. Table 1).”**

**Second round of review**

**Reviewer 1**

The authors have successfully addressed my concerns.

**Reviewer 2**

The authors have addressed all of my concerns. The simulations might benefit from using the fecundity estimates of the lines and could use a bit more detail in their description.

**Authors’ response to reviewers**

We did not include different fecundity in the simulations, because fecundity differences between evolved replicate populations will be only important for the first generation, after this, the genotypes with higher or lower fecundity will mix and are generate mosaic genomes with higher and lower fecundity (note that we did not characterize fecundity differences among lines within evolved replicates). To perform such simulation, we would make some assumptions about the genetic architecture of fecundity and which includes difference among individuals from the same evolved replicate and between evolved replicates. With this information available, it may be possible to include fecundity in the simulations. As we do not have information about the genetic architecture of these fecundity differences, we felt that any simulations would be highly contingent on the assumptions of the genetic architecture. Given the large uncertainty, we reasoned that simulations will not be very informative and did not further pursue this suggestion.
